# Relationship between gait speed and physical function in patients with symptomatic peripheral artery disease

**DOI:** 10.6061/clinics/2019/e1254

**Published:** 2019-10-23

**Authors:** Marilia de Almeida Correia, Gabriel Grizzo Cucato, Fernanda Cordoba Lanza, Roger André Oliveira Peixoto, Antonio Eduardo Zerati, Pedro Puech-Leao, Nelson Wolosker, Raphael Mendes Ritti-Dias

**Affiliations:** I Universidade Nove de Julho, Sao Paulo, SP, BR; II Northumbria University, Newcastle Upon Tyne, Northumbria University, Newcastle Upon TyneUKUK; III Departamento de Fisioterapia, Universidade Federal de Minas Gerais (UFMG), Belo Horizonte, MG, BR; IV Hospital das Clinicas HCFMUSP, Faculdade de Medicina, Universidade de Sao Paulo, Sao Paulo, SP, BR; V Instituto do Cancer do Estado de Sao Paulo (ICESP), Hospital das Clinicas HCFMUSP, Faculdade de Medicina, Universidade de Sao Paulo, Sao Paulo, SP, BR; VI Hospital Israelita Albert Einstein, Sao Paulo, SP, BR

**Keywords:** Intermittent Claudication, Six-Minute Walk Test, Physical Fitness, Gait Speed

## Abstract

**OBJECTIVE:**

The aim of the study was to analyze the relationship between gait speed and measurements of physical function in patients with symptomatic peripheral artery disease (PAD).

**METHODS:**

One hundred sixty-nine patients (age 66.6±9.4 years) with symptomatic PAD were recruited. Usual and fast gait speeds were assessed with a 4-meter walk test. Objective (balance, sit-to-stand, handrip strength, and six-minute walk test) and subjective (WIQ - Walking Impairment Questionnaire and WELCH - Walking Estimated-Limitation Calculated by History) measurements of physical function were obtained. Crude and adjusted linear regression analyses were used to confirm significant associations.

**RESULTS:**

Usual and fast gait speeds were significantly correlated with all objective and subjective physical function variables examined (r<0.55, *p*<0.05). In the multivariate model, usual gait speed was associated with six-minute walking distance (β=0.001, *p*<0.001), sit-to-stand test score (β=-0.005, *p*=0.012), and WIQ stairs score (β=0.002, *p*=0.006) adjusted by age, ankle brachial index, body mass index, and gender. Fast gait speed was associated with six-minute walking distance (β=0.002, *p*<0.001), WIQ stairs score (β=0.003, *p*=0.010), and WELCH total score (β=0.004, *p*=0.026) adjusted by age, ankle brachial index, body mass index, and gender.

**CONCLUSION:**

Usual and fast gait speeds assessed with the 4-meter test were moderately associated with objective and subjective measurements of physical function in symptomatic PAD patients.

## INTRODUCTION

Intermittent claudication is the most common symptom of peripheral artery disease (PAD), affecting approximately 2% of the general population (1). Walking impairment has been related to quality of life (2), physical fitness and cardiovascular function in symptomatic PAD patients (3,4). In addition, pain during walking has been considered an important barrier for physical activity practice (5). Therefore, assessment of physical function has been considered a main outcome in patients with symptomatic PAD.

The progressive graded treadmill test and six-minute walk test (6MWT) have been frequently used to assess physical function in patients with symptomatic PAD (6,7). However, the graded treadmill test and six-minute walk test usually require a specific professional (physician or exercise physiologist) and adequate device (treadmill) or specific location (30-meter corridor). In addition, some patients have difficulties performing treadmill exercise (8), and some learning effects can occur, especially in the 6MWT (9). All these factors limit the objective physical function assessment in vascular clinical practice (7).

Gait speed assessed during a 4-meter walk is simple, quick, reproducible (10), and inexpensive and has emerged as an important tool for physical function assessment in the elderly population. In this age group, gait speed is a strong predictor of functional decline (11) and mortality (12). This test has also been used in symptomatic PAD patients, showing associations with functional decline (12) and quality of life (13). Chen et al. (14) observed that gait speed assessed with the 4-meter walk test is a strong predictor of 6MWT performance in a multivariate model, indicating the potential use of this test as an indicator of functional capacity. However, whether gait speed can be used as a stand-alone indicator of physical function is unknown.

In this study, we analyzed the relationship between gait speed assessed with a 4-meter test in two conditions, usual and fast pace, with objective and subjective measurements of physical function in symptomatic PAD patients.

## MATERIAL AND METHODS

### Experimental approach to the problem

To analyze the relationship between gait speed and physical function in a cross-sectional study, we recruited patients with PAD and symptoms of intermittent claudication. The sample size to achieve significance in correlations ≥0.25, considering an alpha error of 0.05 and a power of 80%, was estimated to be 123 patients. The patients were submitted to an assessment of their gait speed through the 4-meter walk test, which was performed in two conditions: usual pace and fast pace. To assess physical function, a 6MWT, handgrip strength test, static balance test and sit-to-stand test were performed. Patients also answered two specific questionnaires (Walking impairment questionnaire - WIQ and Walking Estimated-Limitation Calculated by History - WELCH) to assess their self-reported physical function. Associations between gait speed (usual and fast pace) with objective and subjective measures of physical function were assessed through multiple linear regression analysis.

### Patients

Symptomatic PAD patients were recruited at hospitals in São Paulo, Brazil, between September 2015 and March 2018. The inclusion criteria were as follows: a) aged 50 years or older with presence of intermittent claudication symptoms (Rutherford stages II and III in one or two legs; b) ankle brachial index (ABI) <0.90; c) absence of critical limb ischemia, rest pain, noncompressible vessels, amputated limbs and/or ulcers, and d) absence of pulmonary diseases.

The study was approved by the Institutional Review Board, performed in accordance with the International Ethics Standards, conformed to the Helsinki Declaration, and approved by the Institutional Ethics Committee.

Prior to data collection and participation, patients were informed about the procedures for participation in the study, as well as the risks and benefits, and signed a written informed consent form.

Sociodemographic, comorbidities, and medication information were obtained through a face-to-face interview. Sociodemographic variables included age, sex (male or female), and educational status (incomplete high school, high school or more). History of smoking habits (ex-, current, or never-smoker), obesity (body mass index (BMI) ≥30 kg/m^2^), diabetes (doctor-diagnosed or use of glucose-lowering drugs), hypertension (systolic/diastolic blood pressure ≥140/90 mmHg or use of antihypertensive drugs), dyslipidemia (doctor-diagnosed or use of lipid-lowering drugs), coronary heart disease, heart failure, and cerebrovascular disease (medical history) were obtained. Body weight was measured via a calibrated scale to the nearest 0.1 kilogram, while height was measured with a stadiometer to the nearest 0.01 m. BMI was calculated as body weight divided by height squared in meters.

PAD severity was identified by a single evaluator using the ABI in accordance with guidelines (15). Briefly, ABI was measured as the highest systolic blood pressure in the posterior tibial or dorsalis pedis artery divided by the highest systolic blood pressure in the brachial artery. Blood pressure measurements were recorded in both limbs using a Doppler vascular monitor (Medmega DV160, Brazil) and a mercury sphygmomanometer.

### Gait speed test

To analyze gait speed, we assessed patients on a 4-meter test. Patients performed this test twice (usual and fast gait) over four meters, and the time was recorded using a stopwatch (within 0.1 seconds). To assess the usual gait speed, patients were instructed to “walk to the other end of the course at your usual speed, just as if you were walking down the street to go to the store.” To assess the fast gait speed, patients were instructed to walk as fast as they could until the end of the course. Patients were allowed to use assistive devices if needed, and each participant was timed for two attempts at usual and fast gait speeds. The fastest walk of each pair of attempts (usual and fast) was used for analyses. To describe the gait speed, a ratio between distance (4-meter) and the time that the patient took to complete the course was calculated.

### Objective measurement of physical function

Handgrip strength was obtained using a dynamometer with digital display (CAMRY, USA) that was adjustable and calibrated with a scale from 0 to 100 kgf. Patients remained seated with a slight adduction of the shoulder, the elbow flexed at 90°, and the forearm and wrist in a neutral position. The test was performed in three attempts for both arms. In each attempt, patients performed a maximal voluntary contraction for five seconds, with a 1-minute interval between each attempt.

The standing balance test required patients to maintain for 10 seconds each stance, with their feet placed side by side, semitandem, and in tandem. For each stance, the interviewer first demonstrated the task, then supported patients to maintain their balance while they positioned their feet, asked if they were ready, released the support and began timing. The timing was stopped when participants moved their feet or grasped the interviewer for support or when 10 seconds had elapsed. Scores ranged from 0 to 4 (maximum performance).

The sit-to-stand test required patients to stand from a chair with their arms across their chest, five times, as quickly as possible, the task was timed from the initial sitting position to the final standing position at the end of the fifth stand. The time measured in seconds was the outcome.

Patients also performed an over-ground 6MWT along a 30-m-long corridor as previously described (16). Briefly, patients were encouraged to “walk at their usual pace for six minutes and cover as much ground as possible” and rest if necessary. At the end of each minute, patients received feedback on the elapsed time, and standardized encouragement in the form of statements such as “you’re doing well, keep it up” and “do your best“ were utilized. The total walked distance was defined as the maximum distance completed by the patient at the end of six minutes.

### Subjective measurement of physical function—Questionnaires

Subjective physical function was measured by validated versions of the WIQ (17) and WELCH questionnaire (18). The WIQ assesses self-reported ambulatory ability in 3 domains: walking distance, walking speed, and stair climbing, with each domain scored on a 0 to 100 scale, where 0 represents extreme limitation and 100 represents no difficulties walking long distances, walking rapidly, or climbing 3 stair flights, respectively. The WELCH questionnaire (18) is a four-question questionnaire related to the speed at which the patient walks normally. The WELCH score ranges from 0 to 100, with zero indicating a patient who can only walk for 30 seconds when walking slowly and who usually walks much slower than relatives, friends or people of the same age without PAD. A score of 100 refers to a patient who can walk 3 hours or more, even when walking fast, and who usually walks faster than relatives, friends or people of the same age without PAD.

### Statistical analysis

The Gaussian distribution and the homogeneity of variance of the data were analyzed by Shapiro-Wilk and Levene tests. Associations between gait speed (usual and fast pace) with physical function (balance, sit-to-stand test, handgrip strength and 6MWT) and subjective physical function (WIQ and WELCH) were assessed through multiple linear regression analysis. Within these models, variables with known associations to either dependent or independent variables were chosen *a priori* and included as covariates to the regression model. Covariates included age, ABI, BMI, and gender. A residual analysis was performed with homoscedasticity examined using graphical analysis (scatter plot) and the Cook-Weisberg test. Multicollinearity was assessed assuming a variance inflation factor below 5.0. The unstandardized coefficients (standard error), coefficient values, and significance were determined in the regression models. All analyses were performed using IBM SPSS Statistics, version 20.0, and the level of significance was set at *p*<0.05 (2-tailed).

## RESULTS

One hundred sixty-nine patients were included in the study, and their clinical characteristics and objective and subjective physical function are shown in Table 1. The mean ABI reflects moderate severity of the disease (ABI: 0.58±0.16). Most of the patients presented with associated risk factors such as hypertension (85%), dyslipidemia (78%) and diabetes mellitus (52%).

Table 2 shows the correlations between gait speed (4-meter usual and fast pace) tests with objective and subjective measurements of physical function. Usual and fast gait speeds were significantly correlated with all objective and subjective physical function variables. There were no correlations between usual gait speed and BMI (*p*>0.417) or between the 4-meter tests and ABI (*p*>0.236). Fast gait speed was not correlated with age (*p*=0.064).

Table 3 shows the results of the adjusted linear regression analysis for the association between gait speed (4-meter usual and fast pace) and objective and subjective measurements of physical function. After adjustments for age, ABI, BMI and gender, the usual gait speed presented associations with the 6MWT (β=0.001, *p*<0.001), sit-to-stand test (β=-0.005, *p*=0.012), and WIQ stairs (β=0.002, *p*=0.006) scores. After adjustments for age, ABI, BMI, and gender, the fast gait speed was associated with the 6MWT (β=0.002, *p*<0.001), WIQ stairs (β=0.003, *p*=0.010), and WELCH total (β=0.004, *p*=0.026) scores.

## DISCUSSION

The main findings of this study were that usual and fast gait speeds were moderately associated with objective and subjective measurements of physical function in symptomatic PAD patients. After adjusting for age, ABI, BMI, and gender, the usual gait speed was associated with the 6MWT, sit-to-stand test and WIQ stairs scores, while the fast gait speed was associated with the 6MWT, WIQ stairs and WELCH total scores.

Despite the potential utility of the 4-meter gait speed test in symptomatic patients with PAD, little information is available regarding the differences between usual or fast gait speed assessments. A previous study showed that fast gait speed is a better predictor of functional decline than usual gait speed, not only in patients with PAD with severe and mild-to moderate intermittent claudication symptoms but also in their peers without the disease (12). In the present study, both modes of gait speed assessments were associated with the 6MWT, with similar magnitude (usual gait: β=0.404 and fast gait: β=0.406).

In the elderly population, age-related loss of strength and muscle mass are one factor causing a decline in gait speed (19-21). In PAD patients, a correlation between leg strength and walking capacity has been reported (2), and the changes in walking capacity with resistance training were related to changes in strength (22). Therefore, assessments of muscle strength are important for patients with PAD. Usual gait speed was related to scores on the sit-to-stand test and WIQ stairs, which are highly dependent on muscle strength. These results expand our current knowledge, indicating that in addition to the 6MWT, gait speed assessed with a 4-meter walk test can also provide information regarding lower limb strength.

The results of the present study indicated that only fast gait speed was associated with WELCH score, a score on a specific questionnaire for PAD patients that accesses walking impairment. In contrast to other questionnaires, the WELCH questionnaire is based on comparisons of peers of the same age. A similar or greater ambulation compared to that of peers is indicated by a higher score in the questionnaire. The association between fast gait speed and WELCH score reveals that the patients who walk faster believe their gait speed to be similar to or better than that of their peers. This association reveals that fast gait speed assessment can also provide information regarding the self-perception of the walking impairment caused by PAD, a relevant clinical outcome for these patients.

From a practical point of view, the results of this study indicated that in symptomatic PAD patients, usual and fast gait speeds assessed with a 4-meter walk test are related to different components of physical function. However, the observed relationships were lower than r=0.55, indicating a coefficient of explanation lower than 30%. Therefore, despite the significant correlations, gait speed assessed with the 4-meter walk test is not an adequate substitute for the objective and subjective functional tests.

This study presented some limitations. The main variable of the present study was obtained with a manual stopwatch (within 0.1 seconds). The sample included only patients with symptomatic PAD, and these results cannot be extrapolated to other stages of the disease, such as asymptomatic patients and critical limb ischemia.

In conclusion, the usual and fast gait speeds assessed with the 4-meter test were moderately associated with objective and subjective measurements of physical function in symptomatic PAD patients. Therefore, despite the significant correlations, gait speed assessed with the 4-meter walk test cannot be a substitute for objective and subjective functional tests.

## AUTHOR CONTRIBUTIONS

Correia MA was responsible for the data collection, data analysis and critical review. Cucato GG was responsible for the study design and critical review. Lanza FC was responsible for the critical review. Peixoto RAO was responsible for the data collection and critical review. Zerati AE, Puech-Leao P, Wolosker N and Ritti-Dias RM were responsible for the study design and critical review.

## Figures and Tables

**Table 1:** Characteristics of the intermittent claudication patients included in the study.

Variables	Values (n=169)
Gender, % men	69
Age, years	66.6±9.4
Body mass index, kg/m^2^	27.6±4.7
Ankle brachial index	0.58±0.16
**Risk factors**	
Diabetes mellitus, %	52
Hypertension, %	85
Dyslipidemia, %	78
Obesity, %	27
Coronary artery disease, %	34
**Functional capacity**	
Usual gait speed, m/s	0.99±0.23
Fast gait speed, m/s	1.53±0.48
Claudication onset distance, m	135±74
Six-minute walk distance, m	338±83
Balance test, score	3.3±1.0
Chair stand test, s	15.5±7.1
Handgrip strength, kgf	32.6±11.8
**Specific PAD questionnaires**	
WIQ distance, score	23.1±21.0
WIQ speed, score	23.5±15.2
WIQ stairs, score	31.3±24.6
WELCH total, score	28.3±19.6

**Table 2:** Correlations between 4-meter test speed with objective and subjective measurements of physical function in symptomatic peripheral artery disease patients (n=169).

Variables	4-meter test speed			
	Usual gait r (*p*)		Fast gait r (*p*)	
Age	-0.250	(0.001)	-0.142	(0.064)
Body mass index	-0.063	(0.417)	-0.059	(0.448)
Ankle brachial index	0.063	(0.414)	0.091	(0.236)
Claudication onset distance	0.340	(<0.001)	0.353	(<0.001)
Six-minute walking distance	0.552	(<0.001)	0.537	(<0.001)
Balance test	0.194	(0.011)	0.193	(0.011)
Sit-to-stand test	-0.352	(<0.001)	-0.298	(<0.001)
Handgrip strength	0.314	(<0.001)	0.279	(<0.001)
WIQ distance	0.346	(<0.001)	0.343	(<0.001)
WIQ speed	0.291	(<0.001)	0.312	(<0.001)
WIQ stairs	0.328	(<0.001)	0.314	(<0.001)
WELCH total	0.236	(0.002)	0.343	(<0.001)

*p* – level of significance; r – Pearson correlation coefficient; WELCH – Walking Estimated-Limitation Calculated by History; WIQ – Walking Impairment Questionnaire.

**Table 3:** Adjusted associations between 4-meter gait speed test speed with objective and subjective measurements of physical function in symptomatic peripheral artery disease patients (n=169).

Dependent variable	Independent variables	**β** (EP)	b	*p*
Usual gait speed	Six-minute walking distance	0.001 (0.001)	0.404	<0.001
	Sit-to-stand test	-0.005 (0.002)	-0.162	0.012
	WIQ stairs	0.002 (0.001)	0.172	0.006
Fast gait speed	Six-minute walking distance	0.002 (0.001)	0.406	<0.001
	WIQ stairs	0.003 (0.001)	0.172	0.010
	WELCH total	0.004 (0.002)	0.160	0.026

β (EP) – Regression coefficient (error-standard); b – Standardized coefficients; *p –* level of significance; WELCH – Walking Estimated-Limitation Calculated by History; WIQ – Walking Impairment Questionnaire; Adjusted by age, ankle brachial index, body mass index, and gender.
